# Histidine biosynthesis, its regulation and biotechnological application in *Corynebacterium glutamicum*

**DOI:** 10.1111/1751-7915.12055

**Published:** 2013-04-25

**Authors:** Robert K Kulis-Horn, Marcus Persicke, Jörn Kalinowski

**Affiliations:** Centrum für Biotechnologie, Universität BielefeldUniversitätsstraße 27, 33615, Bielefeld, Germany

## Abstract

l-Histidine biosynthesis is an ancient metabolic pathway present in bacteria, archaea, lower eukaryotes, and plants. For decades l-histidine biosynthesis has been studied mainly in *Escherichia coli* and *Salmonella typhimurium*, revealing fundamental regulatory processes in bacteria. Furthermore, in the last 15 years this pathway has been also investigated intensively in the industrial amino acid-producing bacterium *Corynebacterium glutamicum*, revealing similarities to *E. coli* and *S. typhimurium*, as well as differences. This review summarizes the current knowledge of l-histidine biosynthesis in *C. glutamicum*. The genes involved and corresponding enzymes are described, in particular focusing on the imidazoleglycerol-phosphate synthase (HisFH) and the histidinol-phosphate phosphatase (HisN). The transcriptional organization of *his* genes in *C. glutamicum* is also reported, including the four histidine operons and their promoters. Knowledge of transcriptional regulation during stringent response and by histidine itself is summarized and a translational regulation mechanism is discussed, as well as clues about a histidine transport system. Finally, we discuss the potential of using this knowledge to create or improve *C. glutamicum* strains for the industrial l-histidine production.

## Introduction

*Corynebacterium glutamicum* is a well-established microorganism for biotechnological applications. Although it has been engineered for the production of various fine chemicals like succinate (Litsanov *et al*., 2012) or isobutanol (Blombach *et al*., 2011), it is still mainly employed for the production of l-amino acids (Becker and Wittmann, 2012). The most important amino acids are l-glutamate (flavour enhancer) and l-lysine (feed additive) based on production scales (Becker and Wittmann, 2011). Furthermore, there are also efforts to create efficient producers for other amino acids like l-leucine, l-serine, and l-methionine. These efforts are supported by a detailed insight into the corresponding amino acid biosynthetic pathways and their regulation in *C. glutamicum* and have been summarized in several reviews or book chapters (Eggeling and Bott, 2005; Wendisch, 2007; Blombach and Seibold, 2010; Brinkrolf *et al*., 2010). However, to date there is no review available about l-histidine biosynthesis and its regulation in this amino acid-producing microorganism. Here, we intend to summarize the current knowledge on histidine biosynthesis, its regulation and attempts for application in *C. glutamicum*. The published data are discussed critically and compared with the knowledge of histidine biosynthesis in *Escherichia coli* and *Salmonella enterica* serovar Typhimurium (*S. typhimurium*), the reference organisms regarding this particular pathway.

### Properties of l-histidine

l-Histidine is one of the 20 standard proteinogenic amino acids present in proteins of all living organisms. In the following, we will use the term histidine instead, meaning its biologically active isomer l-histidine. Its side-chain is an imidazole ring and therefore has aromatic properties. Histidine is the only amino acid whose side-chain can switch from an unprotonated to a protonated state under neutral pH conditions due to the pK_a_ value of 6.0 of its side-chain (Nelson and Cox, 2009). This characteristic enables histidine residues to act as both, a proton acceptor or a proton donor, in many cellular enzymatic reactions (Rebek, 1990; Polgár, 2005).

### The histidine biosynthesis pathway

**Fig. 1:**
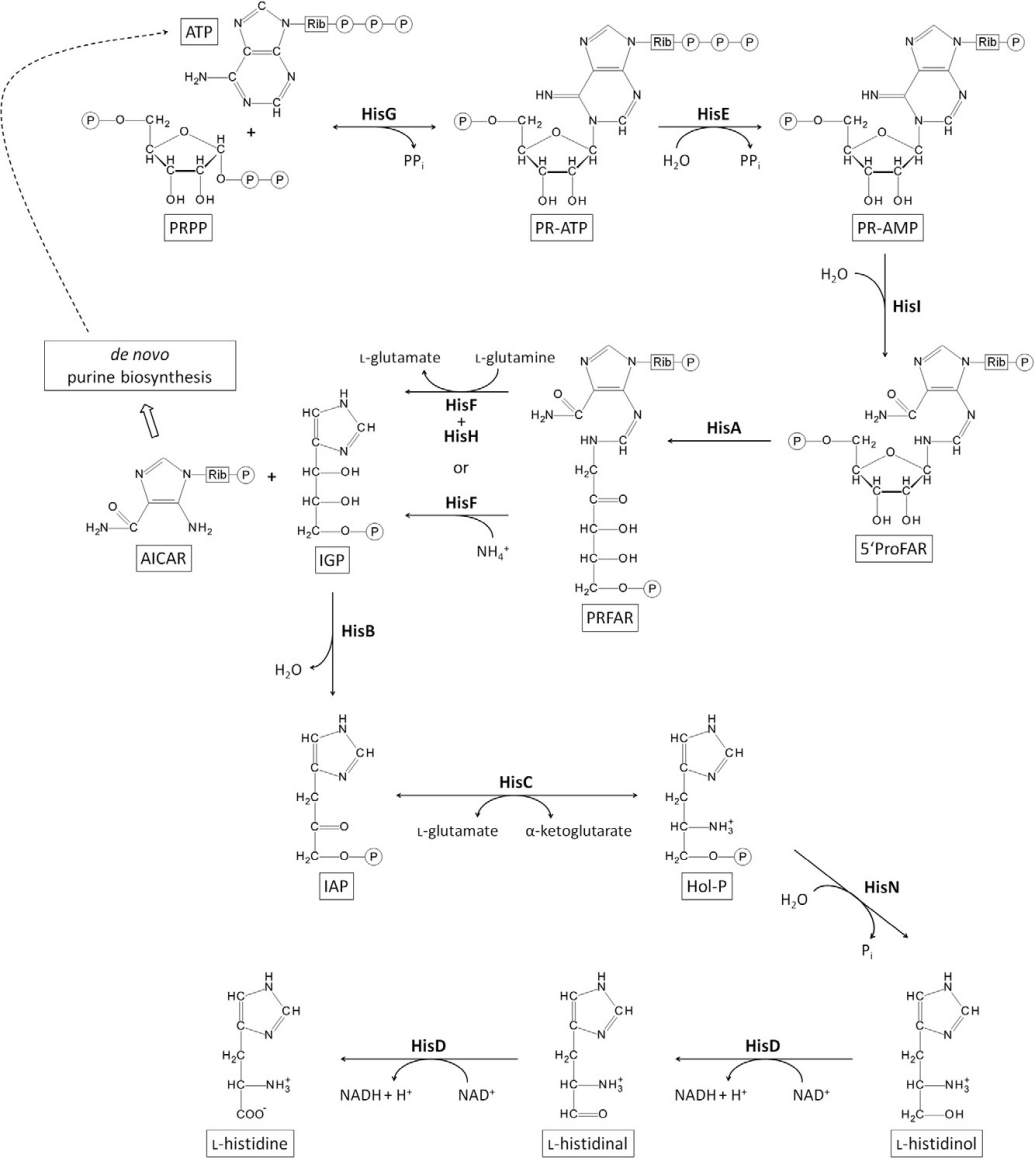
Histidine biosynthetic pathway in *C. glutamicum*. PRPP, phosphoribosyl pyrophosphate; ATP; adenosine triphosphate; PP_i_, pyrophosphate; PR-ATP, phosphoribosyl-ATP; PR-AMP, phosphoribosyl-AMP; 5′ProFAR, 1-(5-phosphoribosyl)-5-[(5-phosphoribosylamino)methylideneamino] imidazole-4 carboxamide; PRFAR, 5-[(5-phospho-1-deoxyribulos-1-ylamino)methylideneamino]-1-(5-phosphoribosyl)imidazole-4-carboxamide; IGP, imidazole-glycerol phosphate; AICAR, 1-(5′-phosphoribosyl)-5-amino-4-imidazolecarboxamide; IAP, imidazole-acetol phosphate; Hol-P, l-histidinol phosphate; P_i_, phosphate; NAD^+^, oxidized nicotinamide adenine dinucleotide; NADH, reduced nicotinamide adenine dinucleotide; HisG, ATP phosphoribosyltransferase; HisE, phosphoribosyl-ATP pyrophosphatase; HisI, phosphoribosyl-AMP cyclohydrolase; HisA, 5′ProFAR isomerase; HisF, synthase subunit of IGP synthase; HisH, glutaminase subunit of IGP synthase; HisB, imidazoleglycerol-phosphate dehydratase; HisC, histidinol-phosphate aminotransferase; HisN, histidinol-phosphate phosphatase; HisD, histidinol dehydrogenase.

Since the late 1950s, the histidine biosynthesis pathway has been studied intensively in different organisms like yeasts, *S. typhimurium*, and *E. coli*. Initially, Ames and Martin elucidated the complete histidine pathway by identifying all metabolic intermediates and the enzymes catalysing the corresponding reactions in *S. typhimurium* (Brenner and Ames, 1971; Martin *et al*., 1971). At that time, last uncertainties remained regarding the reaction steps and intermediates at the interconnection to the pathway of *de novo* purine biosynthesis. These issues were finally elucidated by Klem and Davisson revealing the final number of catalytic reactions and intermediates (Klem and Davisson, 1993). Based on this knowledge, histidine biosynthesis is an unbranched pathway with ten enzymatic reactions, starting with phosphoribosyl pyrophosphate (PRPP) and leading to l-histidine (Fig. 1) (Alifano *et al*., 1996; Stepansky and Leustek, 2006). It turned out early that the histidine pathways of *S. typhimurium* and *E. coli* are identical. Moreover, histidine biosynthesis seems to be conserved in all organisms including archaea (Lee *et al*., 2008), Gram-positive bacteria (Chapman and Nester, 1969), lower eukaryotes (Fink, 1964), and plants (Stepansky and Leustek, 2006). The general histidine pathway and its regulation has already been reviewed in great detail, mainly focusing on *E. coli*, *S. typhimurium*, and plants (Brenner and Ames, 1971; Martin *et al*., 1971; Alifano *et al*., 1996; Winkler, 1996; Stepansky and Leustek, 2006). This work focuses on the histidine biosynthesis, the involved enzymes and its regulation in *C. glutamicum*, since there are some interesting differences in comparison to other organisms.

### *C. glutamicum* as an amino acid producer

*Corynebacterium glutamicum* is a Gram-positive, aerobic, rod shaped, and non-sporulating soil bacterium. It is a member of the genus *Corynebacterium*, family Corynebacteriaceae, order Corynebacteriales (also containing *Mycobacterium* spp.), class Actinobacteria (also containing *Streptomyces* spp. and other filamentous bacteria) (Gao and Gupta, 2012; Goodfellow *et al*., 2012). It was originally isolated in Japan in the late 1950s during a screening for glutamic acid-secreting bacteria (Kinoshita *et al*., 1958). Already the unmodified type strain secretes up to 26 g l^−1^ l-glutamate in minimal medium under biotin-limited conditions and strains improved by classical strain development accumulate more than 100 g l^−1^ of this amino acid in the culture medium (Becker and Wittmann, 2012). Classical strain development played an important role in the beginnings of fermentative amino acid production. Since this technique has reached its limit to further increase productivity, nowadays metabolic engineering is used to further optimize l-glutamate production. At present these engineered strains do not reach the production titres of classical glutamate production strains (Sawada *et al*., 2010). However, there are promising results from metabolic engineering approaches with regard to the production of l-lysine. The implementation of 12 defined genome-based modifications enabled accumulation of 120 g l^−1^ l-lysine in the culture supernatant (Becker *et al*., 2011). These production titres are even higher than those reached with strains created by classical strain development with consecutive rounds of mutagenesis and selection (Becker and Wittmann, 2012). The intensive investigations on l-glutamate and l-lysine biosynthesis pathways and the understanding of their regulation and interconnection to the central metabolism of *C. glutamicum* helped to further improve production strains. Today, about 2.5 million tons of l-glutamate and 1.5 million tons of l-lysine are produced annually by Corynebacteria with estimated growth rates of 6–8% per year (Becker and Wittmann, 2011). There are also several strains available for the production of other amino acids which were created either by classical strain development, by metabolic engineering, or by a combination of both techniques. This includes strains for the production of l-isoleucine, l-tryptophan, l-phenylalanine, l-valine, l-alanine, and l-serine (Becker and Wittmann, 2012).

*Corynebacterium glutamicum* strains suitable for the industrial production of l-histidine have been established by means of combining classical strain development and metabolic engineering. *Corynebacterium glutamicum* mutants resistant to histidine analogues were reported to secrete 6–8 g l^−1^ l-histidine into the culture medium (Araki and Nakayama, 1971). The overexpression of a mutated ATP (adenosine triphosphate) phosphoribosyltransferase which is not inhibited by histidine analogues resulted in a *C. glutamicum* strain accumulating up to 23 g l^−1^ histidine (Mizukami *et al*., 1994). These or similar strains are still used for industrial l-histidine fermentation today (Ikeda, 2003; Becker and Wittmann, 2012).

## Enzymes involved in histidine biosynthesis

### Histidine biosynthesis genes in *C. glutamicum*

*Corynebacterium glutamicum* strain AS019, a derivative of *C. glutamicum* ATCC 13059, was used for the first genetic studies on histidine biosynthesis. The genes *hisA*, encoding the 1-(5-phosphoribosyl)-5-[(5-phosphoribosylamino)methylideneamino]imidazole-4 carboxamide (5′ProFAR) isomerase, and *hisF*, encoding one subunit of the imidazole glycerol phosphate synthase, were identified by complementation of corresponding histidine auxotrophic *E. coli* mutants (Jung *et al*., 1998). The gene *hisH*, coding for the second subunit of imidazole glycerol phosphate synthase (Kim and Lee, 2001), and the genes *hisG* and *hisE*, coding for the ATP phosphoribosyltransferase and phosphoribosyl-ATP pyrophosphatase, respectively, were identified by applying the same method (Kwon *et al*., 2000). The release of the complete genome sequence of the type strain *C. glutamicum* ATCC 13032 in 2003 (Ikeda and Nakagawa, 2003; Kalinowski *et al*., 2003) provided the opportunity for the reconstruction of various metabolic pathways, including histidine biosynthesis. The annotation of the genome led to the identification of genes coding for nine of the 10 enzymatic activities needed for histidine biosynthesis. In addition to the genes *hisAEFGH*, already known from *C. glutamicum* AS019, these were the genes *hisI*, encoding phosphoribosyl-AMP cyclohydrolase, *hisB*, coding for imidazoleglycerol-phosphate dehydratase, *hisC*, coding for histidinol-phosphate aminotransferase, and *hisD*, encoding histidinol dehydrogenase, which catalyses the final two steps of histidine biosynthesis in *C. glutamicum*. However, a gene encoding an enzyme with histidinol-phosphate phosphatase activity has neither been identified by automatic annotation of the genome sequence, nor by heterologous complementation of *E. coli* mutants. In 2006 a random mutagenesis approach using an IS6100-based transposon vector finally identified the gene encoding histidinol-phosphate phosphatase (Mormann *et al*., 2006). The gene was designated *hisN*, since the enzymatic activity is located on the N-terminal part of a bifunctional *hisB* gene product in *S. typhimurium* and *E. coli* (Houston, 1973a; Carlomagno *et al*., 1988). Additionally, the random transposon mutagenesis approach confirmed the involvement of the genes *hisABDEFGI* in histidine biosynthesis. Transposon insertion into either one of these genes resulted in histidine auxotrophy of the corresponding mutants (Mormann *et al*., 2006). Furthermore, participation of the genes *hisBCD* in histidine biosynthesis was again confirmed in complementation experiments with auxotrophic *E. coli* mutants (Jung *et al*., 2009). To sum up, *C. glutamicum* possesses ten histidine biosynthesis genes coding for nine enzymes which catalyse ten enzymatic reactions. This includes one bifunctional enzyme, the histidinol dehydrogenase (*hisD*), and one enzyme consisting of two subunits, the imidazoleglycerol-phosphate synthase (*hisF* and *hisH*). As a part of our own studies, each histidine gene has been deleted individually in *C. glutamicum* (Table 1). As for the transposon mutants, each single in frame deletion of one of the eight genes *hisABCDEFGI* resulted in histidine auxotrophy (R.K. Kulis-Horn, unpubl. obs.), confirming the essentiality of these genes. Interestingly, clear auxotrophies were not found for the deletions of *hisH* and *hisN* (discussed below).

**Table 1:** Histidine biosynthesis genes in *C. glutamicum* ATCC 13032 and the effect of gene disruption on histidine-dependent growth in minimal medium

Gene	Function	Effect of gene disruption	References
*hisG*	ATP phosphoribosyltransferase	auxotrophy	(1), (2), (3)
*hisE*	phosphoribosyl-ATP pyrophosphatase	auxotrophy	(1), (3)
*hisI*	phosphoribosyl-AMP cyclohydrolase	auxotrophy	(1), (3)
*hisA*	5′ProFAR isomerase	auxotrophy	(1), (3)
*hisF*	synthase subunit of IGP synthase	auxotrophy	(1), (3)
*hisH*	glutaminase subunit of IGP synthase	no effect	(3)
*hisB*	imidazoleglycerol-phosphate dehydratase	auxotrophy	(1), (3)
*hisC*	histidinol-phosphate aminotransferase	auxotrophy	(3)
*hisN*	histidinol-phosphate phosphatase	auxotrophy^a^	(1), (3)^a^
*hisD*	histidinol dehydrogenase	auxotrophy	(1), (3)
*cg0911*	putative inositol monophosphatase	no effect	(3)
*impA*	putative inositol monophosphatase	no effect	(3)
*cg2301*	putative antibiotic efflux permease, MFS-type	no effect	(3)
*cg1305*	l-phenylalanine transporter	no effect^b^	(3), (4)

a Growth strongly reduced but still measurable (this study).

b No growth of a Δ*cg1305* Δ*hisG* double mutant in histidine supplemented medium (this study).

(1) Mormann *et al*. (2006): transposon insertion.

(2) Zhang *et al*. (2012): substitution of *hisG* 3′ end with a chloramphenicol resistance gene.

(3) This study: in frame gene deletion.

(4) Zhao *et al*. (2011): in frame gene deletion.

### ATP phosphoribosyltransferase (HisG)

ATP phosphoribosyltransferase (ATP-PRT) catalyses the first step of histidine biosynthesis, the condensation of ATP and PRPP to phosphoribosyl-ATP (PR-ATP) and pyrophosphate (PP_i_) (Alifano *et al*., 1996). ATP phosphoribosyltransferases can be divided into two subfamilies, the *long* and the *short* ATP-PRTs. Enzymes of the *long* subfamily are 280–310 amino acids in length and are present in lower eukaryotes and bacteria, like *E. coli*, *S. typhimurium*, or *Mycobacterium tuberculosis* (Zhang *et al*., 2012). The *short* forms of ATP-PRTs are lacking about 80 amino acids at their C-terminus. They are present in some bacteria, such as *Bacillus subtilis*, *Lactococcus lactis*, and *Pseudomonas aeruginosa* (Bond and Francklyn, 2000). These *short* ATP-PRTs require the presence of the *hisZ* gene product for their catalytic activity (Sissler *et al*., 1999). The HisZ protein has no sequence homology to the C-terminus of *long* ATP-PRTs, but is a paralogue of histidyl-tRNA synthetase (Sissler *et al*., 1999).

With a length of 281 amino acids, ATP-PRT from *C. glutamicum* (HisG*_Cg_*) belongs to the *long* form of ATP-PRTs. Therefore, it is not surprising that the *C. glutamicum* genome lacks a paralogue of the *hisZ* gene. Kinetic parameters of HisG*_Cg_* have been determined recently. The enzyme has a specific activity of 2.19 ± 0.09 μmol min^−1^ mg^−1^, a K_m_ value for PRPP of 0.08 ± 0.01 mM, a K_m_ value for ATP of 0.22 ± 0.02, and a k_cat_ value of 1.91 ± 0.14 s^−1^ (Zhang *et al*., 2012). Comparison of crystal structures and structure-based multiple alignments of ATP-PRTs from bacteria, archaea, and baker's yeast revealed a common 3D structure of ATP-PRTs (Zhang *et al*., 2012). ATP-PRTs have no structural and sequence similarities to other phosphoribosyltransferases, besides the PRPP binding site. Therefore, ATP-PRT is considered a member of the new type IV class of phosphoribosyltransferases (Lohkamp *et al*., 2004; Zhang *et al*., 2012). The crystal structure of HisG*_Cg_* is not available yet. However, a homology model based on the 3D structure of ATP-PRT from *M. tuberculosis* (HisG*_Mt_*) (62% sequence identity and 89% sequence similarity) revealed an almost identical structure to HisG*_Cg_* (Zhang *et al*., 2012). Knowledge about the 3D structure of HisG*_Mt_* is therefore most likely also true for HisG*_Cg_*. According to the predicted structure model, HisG*_Cg_* is a L-shaped monomer composed of three distinct domains (Zhang *et al*., 2012). The first two domains form the catalytic core. The active site is located in a cleft between these two domains. The third domain is able to bind histidine and is therefore regarded as the regulatory domain (Cho *et al*., 2003; Zhang *et al*., 2012). The native HisG enzyme from *E. coli* and *S. typhimurium* is in equilibrium between a dimeric and hexameric form (Winkler, 1996). Gel filtration experiments with purified HisG*_Cg_* confirmed this quaternary structure in *C. glutamicum* (Zhang *et al*., 2012). ATP-PRT is subject to feedback inhibition and its activity is also influenced by additional factors such as enzyme concentration or the energy status of the cell (Araki and Nakayama, 1974; Zhang *et al*., 2012). Since, the regulation of ATP-PRT is of great importance it will be discussed in more detail below.

### Phosphoribosyl-ATP pyrophosphatase (HisE) and phosphoribosyl-AMP cyclohydrolase (HisI)

Phosphoribosyl-ATP pyrophosphatase catalyses the irreversible hydrolysis of PR-ATP to phosphoribosyl-AMP (PR-AMP) in the second step of histidine biosynthesis. Subsequently, in the third step PR-AMP cyclohydrolase opens the purine ring of PR-ATP releasing 1-(5-phosphoribosyl)-5-[(5-phosphoribosylamino) methylideneamino] imidazole-4 carboxamide (5′ProFAR) (Alifano *et al*., 1996). Both enzymatic activities are carried out by a single polypeptide chain in *E. coli* and *S. typhimurium* (Carlomagno *et al*., 1988). In *C. glutamicum*, the two activities are encoded by separate genes (Kalinowski *et al*., 2003). Bifunctional His(IE) enzymes exist in all eukaryotes and in several unrelated taxonomic bacterial lineages, but are absent in all Actinobacteria (Fani *et al*., 2007). Most likely, bifunctional His(IE) proteins in bacteria are the result of several independent fusion events and horizontal gene transfer (Fani *et al*., 2007). The native bifunctional His(IE) enzymes from *E. coli* and *S. typhimurium* act as dimers (Winkler, 1996). The crystal structure of phosphoribosyl-ATP pyrophosphatase from *M. tuberculosis* (HisE*_Mt_*) was solved and revealed that it also forms a dimer (Javid-Majd *et al*., 2008). The amino acid sequences of HisE*_Cg_* and HisE*_Mt_* share 62% identity and 90% similarity, assuming a very similar structure for both proteins. Based on this deduced 3D structure, native HisE*_Cg_* most likely acts as a dimer, too.

### 5′ProFAR isomerase (HisA)

The fourth step of histidine biosynthesis is performed by 5′ProFAR isomerase. This enzyme catalyses an internal redox reaction converting 5′ProFAR to 5-[(5-phospho-1-deoxyribulos-1-ylamino)methylideneamino]-1-(5-phosphoribosyl)imidazole-4-carboxamide (PRFAR) (Alifano *et al*., 1996). The native enzymes from *E. coli* and *S. typhimurium* act as monomers (Winkler, 1996). The crystal structure of 5′ProFAR isomerase from *M. tuberculosis* (PriA*_Mt_*) encoded by the *priA* gene was solved recently (Due *et al*., 2011). Interestingly, PriA*_Mt_* is also involved in tryptophan biosynthesis due to its phosphoribosylanthranilate isomerase activity. So far it cannot be excluded that 5′ProFAR isomerase from *C. glutamicum* (HisA*_Cg_*) is also involved in tryptophan biosynthesis. However, deletion of *hisA* resulted in histidine auxotrophy only (R.K. Kulis-Horn, unpubl. obs.), indicating that *C. glutamicum* must at least possess one additional gene coding for a phosphoribosylanthranilate isomerase. This enzyme activity is most likely exerted by the *trp(CF)* gene product, already annotated as a bifunctional phosphoribosylanthranilate isomerase/indoleglycerolphosphate synthase in *C. glutamicum* (Kalinowski *et al*., 2003). Nevertheless, the 3D structure of the bifunctional PriA*_Mt_* enzyme, exhibiting 61% identity and 89% similarity on amino acid level, allows a deeper insight into the structure of 5′ProFAR isomerase from *C. glutamicum* (HisA*_Cg_*). Based on these data, native HisA*_Cg_* most likely acts as a monomer with an (α/β)_8_ barrel fold. [Corrections added on 09 October 2013, after first online publication: In the paragraph above, occurrences of the gene name “pirA” are now amended to “priA”.]

### Imidazoleglycerol-phosphate synthase (HisFH)

The fifth step of histidine biosynthesis is the conversion of PRFAR to the next histidine intermediate imidazole-glycerol phosphate (IGP) and the byproduct 1-(5′-phosphoribosyl)-5-amino-4-imidazolecarboxamide (AICAR), an intermediate of *de novo* purine biosynthesis (Alifano *et al*., 1996). Glutamine is used as nitrogen donor in this amination step releasing glutamate (Smith and Ames, 1964). Mutations in either *hisH* or *hisF* result in histidine auxotrophy of *S. typhimurium* (Hartman *et al*., 1960). These genes were later linked to the fifth step of histidine biosynthesis, although both were initially assumed to code for independent enzymes catalysing different steps in the conversion of PRFAR to IGP and AICAR (Smith and Ames, 1964). The exact role of *hisF* and *hisH* gene products remained elusive for many years. It was finally demonstrated for *hisF* and *hisH* of *E. coli* that the two gene products act as a stable 1:1 dimeric complex which constitutes the IGP synthase holoenzyme (Klem and Davisson, 1993).

*Corynebacterium glutamicum* also possesses *hisF* and *hisH genes*. They exhibit 44% and 38% identity on amino acid level compared with enzymes from *E. coli* respectively. A genomic DNA fragment containing both genes from *C. glutamicum* AS019 was able to complement histidine auxotrophic *hisF* and *hisH E. coli* mutants, demonstrating that these two gene products have the same catalytic activities in both organisms (Jung *et al*., 1998; Kim and Lee, 2001). In accordance with these results, the deletion of *hisF* resulted in histidine auxotrophy in *C. glutamicum*. The deletion of *hisH*, however, did not have any effect on the growth behaviour of the mutant grown in minimal medium (R.K. Kulis-Horn, unpubl. result). This finding is also accordant with the results from the transposon mutagenesis approach where a transposon insertion in *hisH* was not observed in any of the histidine auxotrophic mutants (Mormann *et al*., 2006). There are different possible explanations for this surprising growth behaviour of the Δ*hisH* mutant on minimal medium. (1) The *hisH* gene in *C. glutamicum* might be wrongly annotated and another gene has the true *hisH* gene function. (2) There is a *hisH* paralogue which complements the gene function. (3) Unlike in *E. coli* and *S. typhimurium*, *hisH* is not essential for histidine biosynthesis in *C. glutamicum*.

Concerning hypotheses (1) and (2): There are no further genes within the genome of *C. glutamicum* encoding proteins with considerable sequence similarities to HisH (glutaminase subunit of IGP synthase). The two best blast hits are with *pabAB* (*cg1134*) and *trpG* (*cg3360*). The *pabAB* gene encodes a para-aminobenzoate synthase, an enzyme involved in folic acid biosynthesis (Stolz *et al*., 2007), and *trpG*, encoding the second subunit of anthranilate synthase, is involved in tryptophan biosynthesis (Heery and Dunican, 1993). It is known from studies with other organisms that these enzymes exhibit glutamine amidotransferase activity, which is also the reaction performed by HisH (Crawford and Eberly, 1986; Viswanathan *et al*., 1995). In theory, these two enzymes could take over the enzymatic activity of HisH. But this scenario seems rather unlikely, since it was demonstrated for IGP-synthase from *E. coli* that two perfectly matching HisF (synthase subunit of IGP synthase) and HisH monomers are needed for glutaminase acivity of HisH and channelling of ammonia to the catalytic centre of HisF (Klem *et al*., 2001; Amaro *et al*., 2005).

Concerning hypothesis (3): *E. coli* HisF is able to perform the fifth step of histidine biosynthesis without HisH activity *in vitro* in the presence of unphysiologically high ammonia concentrations and pH > 8 (Smith and Ames, 1964; Klem and Davisson, 1993). The HisH activity is only needed if glutamine is the only nitrogen donor in the *in vitro* reaction, since this subunit of the IGP synthase exhibits a glutamine amidotransferase activity (Klem and Davisson, 1993). However, glutamine seems to be the true nitrogen donor *in vivo*. Mutations in *hisH* result in histidine auxotrophy of *S. typhimurium* and *E. coli* despite the presence of ammonia in the minimal medium (Hartman *et al*., 1960). On the contrary, a *C. glutamicum* Δ*hisH* mutant still grows in ammonia containing minimal medium (R.K. Kulis-Horn, unpubl. obs.). The IGP synthase from *C. glutamicum* seems to have different properties than the enzymes from *S. typhimurium*, *E. coli*, and other species reported. The most probable explanation for this phenomenon is an ammonia-dependent substrate amination activity of HisF*_Cg_ in vivo* (Fig. 1). Our findings support this theory, since *hisF_Cg_* is able to complement both, a *hisF* and a *hisH* deletion, in *E. coli* (R.K. Kulis-Horn and P. Humbert, unpubl. obs.). The other possibility, a glutamine amidotransferase activity already present in the HisF protein like observed in the monomeric IGP synthase HIS7 from *Saccharomyces cerevisiae* (Kuenzler *et al*., 1993), seems unlikely. HisF*_Cg_* is only of the size of HisF*_Ec_* and does not exhibit any sequence similarities to known amidotransferases. The overexpression of *hisH_Cg_* is able to complement a *hisH* deletion in *E. coli*, demonstrating that the *hisH*_Cg_ gene product is functional though not needed in *C. glutamicum* (Jung *et al*., 1998). So far, no other IGP synthase has been reported being able to catalyse the fifth step of histidine biosynthesis without glutamine amidotransferase activity *in vivo*. These findings are very interesting especially in the view of the biotechnological application of *C. glutamicum* as histidine producer, since histidine production in this organism seems to be independent of glutamine biosynthesis.

### Imidazoleglycerol-phosphate dehydratase (HisB)

The imidazoleglycerol-phosphate dehydratase catalyses the sixth step of histidine biosynthesis. The enzyme dehydrates IGP and the resulting enol is then ketonized non-enzymatically to imidazole-acetol phosphate (IAP) (Alifano *et al*., 1996). In *S. typhimurium* and *E. coli* this step is catalysed by a bifunctional enzyme comprising both, the imidazoleglycerol-phosphate dehydratase activity and the histidinol-phosphate phosphatase activity, catalysing the eighth step of biosynthesis (Loper, 1961; Houston, 1973a). In these two organisms the bifunctional enzyme is encoded by the *his(NB)* gene, comprising phosphatase activity at the N-terminus of the encoded protein and dehydratase activity at the C-terminus (Houston, 1973b; Rangarajan *et al*., 2006). There is evidence that this bifunctional *his(NB)* gene results from a rather recent gene fusion event in the γ-proteobacterial lineage (Brilli and Fani, 2004). In eukaryotes, archaea and most bacteria the two activities are encoded by separate genes (Fink, 1964; le Coq *et al*., 1999; Lee *et al*., 2008). This is also true for *C. glutamicum*, with IGP dehydratase being encoded by *hisB* and histidinol-phosphate phosphatase by *hisN* (Mormann *et al*., 2006; Jung *et al*., 2009).

### Histidinol-phosphate aminotransferase (HisC)

The seventh step of histidine biosynthesis is the transamination of IAP to l-histidinol phosphate (Hol-P) using glutamate as amino group donor (Alifano *et al*., 1996). This step is catalysed by the pyridoxal 5′-phosphate (PLP) dependent histidinol-phosphate aminotransferase in *C. glutamicum* (Marienhagen *et al*., 2008). Like HisC from *E. coli* and *S. typhimurium* (Winkler, 1996), native HisC*_Cg_* acts as a dimer (Marienhagen *et al*., 2008). Kinetic parameters of HisC*_Cg_* were determined only for the back-reaction converting Hol-P and α-ketoglutarate into IAP and l-glutamate. The enzyme exhibits a K_m_ value for Hol-P of 0.89 ± 0.1 mM, a k_cat_ value of 1.18 ± 0.1 s^−1^ and a specific activity of 2.8 μmol min^−1^ mg^−1^ (Marienhagen *et al*., 2008). Interestingly, HisC*_Cg_* shows also activity with the precursors of leucine and aromatic amino acids in *in vitro* assays, but the K_m_ values are two orders of magnitude higher compared with those observed with the histidine precursor and HisC*_Cg_* does not contribute to aromatic amino acid synthesis *in vivo* (Marienhagen *et al*., 2005; 2008). The crystal structure of HisC*_Cg_* has been solved revealing a three-domain structure of the monomer, with a N-terminal arm, a large PLP binding domain, and a small C-terminal domain (Marienhagen *et al*., 2008). HisC*_Cg_* dimerization occurs via extensive hydrophobic interactions and 24 intersubunit hydrogen bonds with the N-terminal arm contributing significantly to the intersubunit interface (Marienhagen *et al*., 2008). The active sites are made up almost exclusively of residues within one subunit, but the tight packing of the dimer shields the active sites from the solvent (Marienhagen *et al*., 2008). Site-directed mutagenesis experiments highlighted the importance of the conserved residue Tyr21 for Hol-P substrate specificity and Asn99 for the orientation of the cofactor PLP inside the active centre (Marienhagen *et al*., 2008). Recently, the structure of histidinol-phosphate aminotransferase from *M. tuberculosis* (HisC2*_Mt_*) has also been published (Nasir *et al*., 2012). Interestingly, in *M. tuberculosis* two genes (*hisC1* and *hisC2*) are annotated encoding Hol-P aminotransferases (Camus *et al*., 2002). The first gene is clustered together with other histidine biosynthesis genes in the same order as in *C. glutamicum*. The second gene, however, is monocistronic and located in the genome distant from other *his* genes. The deduced amino acid sequence of *hisC2* from *M. tuberculosis* is most similar to the aromatic amino acid aminotransferase encoded by *aroT* (*cg0267*) in *C. glutamicum*. HisC*_Cg_* and AroT*_Cg_* both exhibit high sequence similarity to Hol-P aminotransferases (McHardy *et al*., 2003). Whereas HisC*_Cg_* is most similar to aminotransferases being exclusively involved in histidine biosynthesis, AroT*_Cg_* is more similar to aminotransferases with a broader substrate spectrum being involved in histidine but also aromatic amino acid biosynthesis (McHardy *et al*., 2003). Enzyme assays with purified AroT*_Cg_* demonstrated its involvement in synthesis of the aromatic amino acids tyrosine and phenylalanine (Marienhagen *et al*., 2005). Its function in histidine biosynthesis was not assayed. However, since the presence of the *aroT* gene is not able to prevent histidine auxotrophy in a *hisC* deletion mutant of *C. glutamicum* (R.K. Kulis-Horn, unpubl. obs.) it is very likely that *aroT_Cg_* does not encode a Hol-P aminotransferase. The structure of the protein crystallized by Nasir and colleagues (2012) therefore does not give deeper insight into the 3D structure of Hol-P aminotransferase from *C. glutamicum*, but rather into the structure of AroT*_Cg_*.

### Histidinol-phosphate phosphatase (HisN)

During the eighth step of histidine biosynthesis Hol-P is dephosphorylated to l-histidinol. In *E. coli* and *S. typhimurium* this reaction is catalysed by a bifunctional enzyme comprising both, the Hol-P phosphatase activity and the IGP dehydratase activity catalysing the sixth step of the biosynthesis (see above). In *C. glutamicum* both activities are encoded by two genes, *hisB* encoding IGP phosphatase (Jung *et al*., 2009) and *hisN* encoding Hol-P phosphatase (Mormann *et al*., 2006). IGP phosphatases seem to be derived from a common ancestor in all organisms. But there is a difference in the origin of the Hol-P phosphatases being part of a bifunctional enzyme and those being encoded by a separate gene (Brilli and Fani, 2004). Bifunctional Hol-P phosphatases are members of the HAD family of the DDDD-superfamily of phosphatases. However, the monofunctional ones, present in, e.g. *B. subtilis* and *L. lactis*, belong to the PHP-superfamily (Brilli and Fani, 2004). The *hisN* gene product from *C. glutamicum* neither exhibits characteristics of the DDDD- nor the PHP-superfamily, thus representing a new class of Hol-P phosphatases. HisN*_Cg_* is grouped into the family of bacterial-like inositol monophosphatases (IMPase), a member of the FIG-superfamily, based on search results within the Conserved Domain Database (Marchler-Bauer *et al*., 2010). Homologues of the monofunctional HisN from *C. glutamicum* can be found predominately in high GC Gram-positive bacteria (blastp). Almost all taxonomical orders of the class Actinobacteria contain genera with HisN homologues, including the Actinomycetales, Corynebacteriales, with the important families Corynebacteriaceae and Mycobacteriaceae, Frankiales, Micrococcales and Streptomycetales (data not shown). Due to the high sequence similarity to IMPase it is hard to decide on the basis of the sequence alone if a *hisN* homologue encodes a Hol-P phosphatase. Four genes exhibiting high sequence homology to *hisN_Cg_* are already present in the genome of *C. glutamicum*. These genes are *cg0911*, *cg2090* (*suhB*), *cg2298* (*impA*), and *cg0967* (*cysQ*), all encoding proteins with domains typical of inositol monophosphatases (Mormann *et al*., 2006).

Deletion of *hisN* was reported to result in histidine auxotrophy in *C. glutamicum* (Mormann *et al*., 2006). Contrary to this, Jung and colleagues (2009) reported the cloning and identification of all *C. glutamicum his* genes without mentioning the *hisN* gene and evidence for the need of such a gene by performing complementation studies with histidine auxotrophic *E. coli* mutants. This discrepancy can be explained by the *E. coli* mutants used in the study of Jung and colleagues (2009). The *E. coli hisB463* mutant used had a deletion of the distal part of the *hisB* gene encoding the imidazoleglycerol-phosphate dehydratase activity, but the histidinol phosphate phosphatase activity is not affected in this strain (Struhl and Davis, 1977). We observed a strongly impaired growth of a *C. glutamicum* Δ*hisN* mutant on minimal medium, but no complete histidine auxotrophy, indicating the existence of at least one more gene encoding a protein with HisN activity (R.K. Kulis-Horn, unpubl. obs.). Most likely, one of the four *hisN_Cg_* homologues present in *C. glutamicum* is able to partially complement the *hisN* deletion.

### Histidinol dehydrogenase (HisD)

The last two steps of histidine biosynthesis are catalysed by a single enzyme. l-Histidinol is first oxidized by histidinol dehydrogenase to l-histidinal, which is further oxidized to l-histidine (Alifano *et al*., 1996). Both steps are catalysed by the same enzyme to prevent the decomposition of the unstable l-histidinal intermediate (Görisch and Hölke, 1985) and two molecules NAD^+^ (oxidized nicotinamide adenine dinucleotide) are reduced during the reaction (Adams, 1954). The native HisD enzyme from *S. typhimurium* (HisD*_St_*) acts as a homodimer and both subunits are linked by disulfide bridges (Eccleston *et al*., 1979). HisD*_St_* is Zn^2+^ dependent (Grubmeyer *et al*., 1989). Native histidinol dehydrogenase from *M. tuberculosis* (62% identity, 83% similarity to HisD from *C. glutamicum*) also acts as a homodimer and is metal dependent (Nunes *et al*., 2011). However, it remaines uncertain if Zn^2+^ or rather Mn^2+^ is the preferred metal ion. Nunes *et al*. also performed molecular homology modelling of HisD*_Mt_* employing the crystal structure of histidinol dehydrogenase from *E. coli* (Barbosa *et al*., 2002) as template. Enzymes from both organisms have a very similar structure. Each homodimer comprises two identical active sites located at the interface of both subunits. Residues from both subunits form the binding sites for l-histidinol and the metal ion, whereas NAD^+^ binds only to residues from one subunit (Barbosa *et al*., 2002; Nunes *et al*., 2011). A Bi-Uni Uni-Bi ping-pong reaction mechanism was proposed for HisD*_Mt_*. l-Histidinol binds first, followed by NAD^+^. NADH+H^+^ is released while l-histidinal stays enzyme-bound. Then the second NAD^+^ binds and is reduced, again releasing NADH+H^+^ and finally l-histidine (Nunes *et al*., 2011). This reaction mechanism most probably also reflects the HisD*_Cg_* reaction mechanism.

## Transcriptional organization of the histidine biosynthesis genes

The histidine gene cluster of *S. typhimurium* and *E. coli* was one of the model gene clusters leading to the development and approval of the operon theory (Alifano *et al*., 1996). In these two organisms all eight histidine biosynthesis genes are part of one operon and therefore trancribed and regulated as a single unit (Martin, 1963b; Fink and Martin, 1967; Carlomagno *et al*., 1988). This concentration of all histidine biosynthesis genes at one locus seems not to be the rule but rather an exception and restricted to the enterobacteria, since in other bacteria *his* genes are more scattered throughout the genome (Alifano *et al*., 1996).

### Transcriptional organization of histidine genes in *C. glutamicum*

**Fig. 2:**
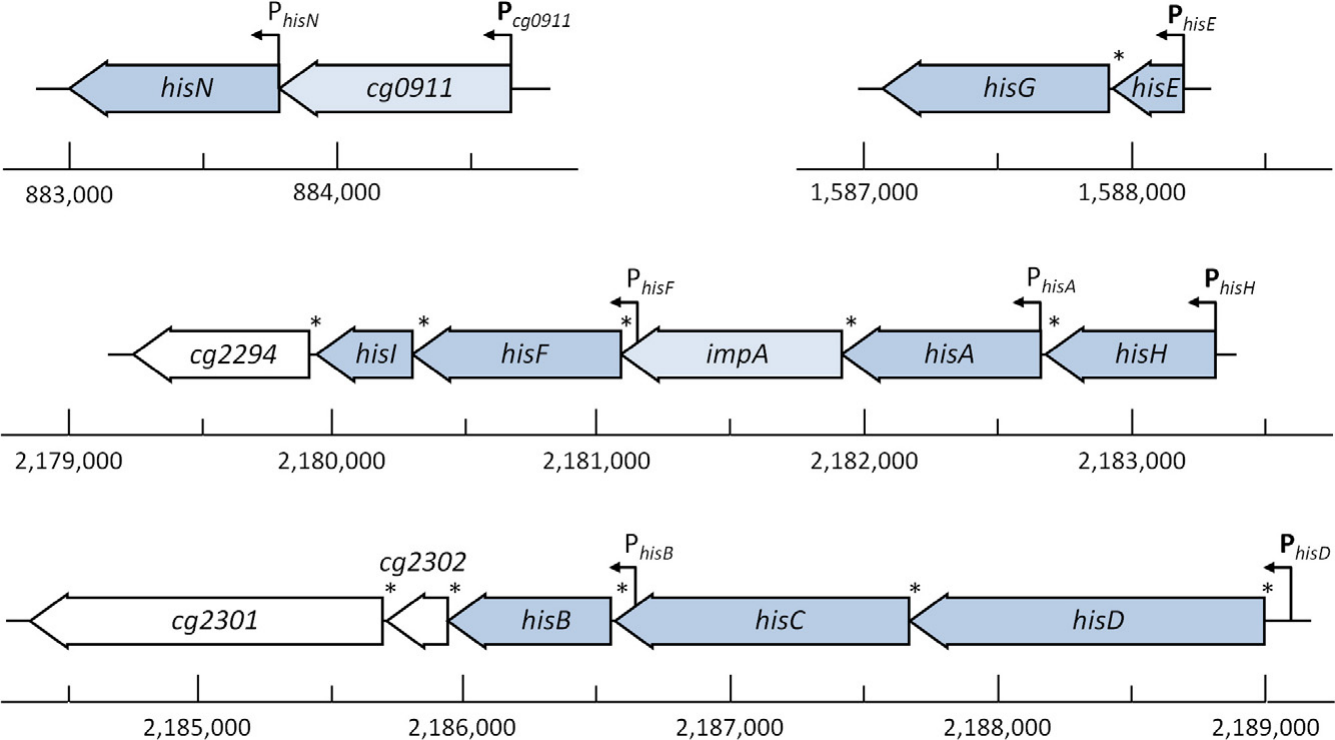
Structure of the four histidine operons in *C. glutamicum*. Canonical histidine biosynthesis genes are depicted in dark blue. Genes shown in light blue exhibit high sequence similarity to *hisN*. Genes shown in white have no apparent function in histidine biosynthesis. Arrows indicate the positions of putative primary and internal promoters. Presence of a SD sequence is marked with an asterisk. The ruler indicates the absolute position within the genome (based on the genome version by Kalinowski *et al*., 2003 RefSeq NC_006958.1).

Jung and colleagues (2009) reported that the histidine genes in *C. glutamicum* AS019 are located and transcribed in two unlinked loci, *hisEG* and *hisDCB-orf1-orf2-hisHA-impA-hisFI*. As this study missed the *hisN* gene, the number of histidine loci increases to three (see above). The genes *orf1* and *orf2* correspond to genes *cg2302* and *cg2301* in *C. glutamicum* ATCC 13032 respectively. The release of the complete genome sequence of *C. glutamicum* (Kalinowski *et al*., 2003) revealed that the *hisN*, *hisGE*, and *hisDCB-cg2302-cg2301-hisHA-impA-hisFI* loci are each separated by several hundred kilobase pairs forming independent transcriptional units (Fig. 2). A closer look is needed to verify the operon structure of the *hisDCB-cg2302-cg2301-hisHA-impA-hisFI* locus. The conclusion that the genes *hisDCB-orf1-orf2-hisHA-impA-hisFI* form one transcriptional unit in *C. glutamicum* AS019 is based on results from RT-PCR analysis (Jung *et al*., 2009). In *C. glutamicum* ATCC 13032 the genes *cg2301* and *hisH* are separated by a 1044 bp non-coding region. In contrast to the results from strain AS019, this large intergenic region implies two independent transcriptional units for *hisDCB-cg2302-cg2301* and *hisHA-impA-hisFI*. Strand specific cDNA sequencing (RNA-Seq) was performed for the whole transcriptome of *C. glutamicum* ATCC 13032 in our group (K. Pfeifer-Sancar, A. Mentz, C. Rückert, and J. Kalinowski, manuscript in preparation). This study enabled for the first time the analysis of transcripts with a single nucleotide resolution for whole pathways in one experiment. The RNA-Seq data revealed transcription of all 10 histidine biosynthesis genes, but it did not give any evidence for transcripts spanning the non-coding region between *cg2301* and *hisH* in *C. glutamicum* ATCC 13032 under all tested conditions (complex and minimal media, various stresses; R.K. Kulis-Horn, unpubl. data). Furthermore, the data showed transcription start sites in front of the *hisD* and the *hisH* gene. It appears that *hisDCB-cg2032-cg2301* and *hisHA-impA-hisFI* represent two unlinked transcriptional units in *C. glutamicum* ATCC 13032 (Fig. 2). The genes *cg2294*, c*g2301* and *cg2302* are of unknown function. The deduced protein sequence of *cg2301* shows characteristics of a permease of the major facilitator superfamily (blastp).

Furthermore, the identification of transcription start sites by RNA-Seq revealed that the operons *cg0911*-*hisN*, *hisEG*, and *hisHA-impA-hisFI-cg2294* are transcribed as leaderless mRNAs, meaning that the start of transcription is identical with the translational start site of the first gene (R.K. Kulis-Horn, unpubl. data). Leaderless transcripts are rarely found in Firmicutes and Proteobacteria, but are common in Actinobacteria where they represent on average 20% of all transcripts (Zheng *et al*., 2011). The *hisDCB-cg2302-cg2301* operon on the other hand comprises a 5′ untranslated region (5′ UTR) with a classical Shine–Dalgarno (SD) sequence. The length of this 5′ UTR was determined by means of primer extension experiments to be 196 nucleotides in *C. glutamicum* AS019 (Jung *et al*., 2009). The RNA-Seq data for *C. glutamicum* ATCC 13032 revealed a shorter 5′ UTR comprising only 93 nucleotides (R.K. Kulis-Horn, unpubl. data). Although the DNA sequence of both *C. glutamicum* strains is identical in this particular region, there is no evidence for a transcription start site in *C. glutamicum* ATCC 13032 corresponding to the position mapped in *C. glutamicum* AS019 (data not shown).

### Promoters

**Fig. 3:**
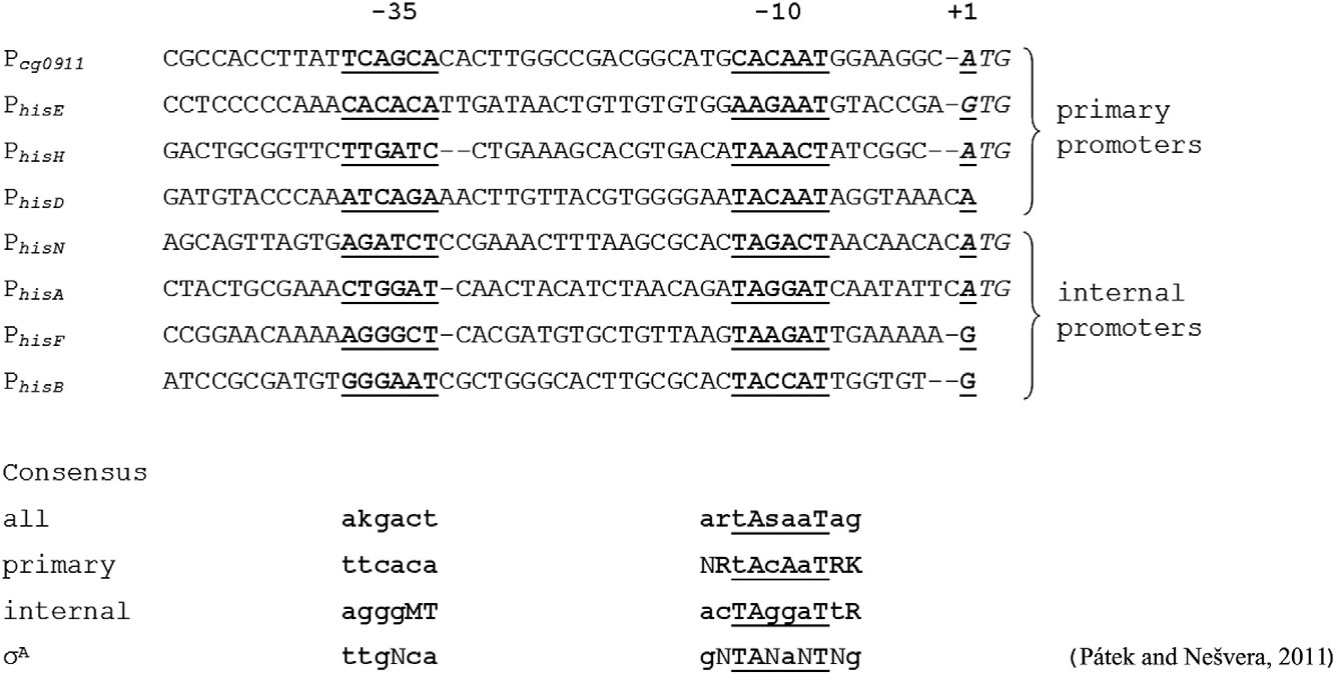
Putative promoter sequences of histidine biosynthesis genes. Transcription start sites (+1) were determined by means of RNA-Seq (K. Pfeifer-Sancar, A. Mentz, C. Rückert, and J. Kalinowski, manuscript in preparation). Putative −10 and −35 boxes are shown in bold and underlined. Dashes indicate gaps of 1–2 nt introduced into the sequence to align the −10 and −35 boxes and the transcription start sites. The start codons are highlighted in italics. The promoter consensus sequences were calculated using either the sequence of all eight promoters, the four primary promoters (P*_cg0911_*, P*_hisE_*, P*_hisH_*, P*_hisD_*), or the four internal promoters (P*_hisN_*, P*_hisA_*, P*_hisF_*, P*_hisB_*). The consensus sequence of sigma factor A (σ^A^) dependent promoters from *C. glutamicum* (Pátek and Nešvera, 2011) is shown in addition. The consensus sequence represents nucleotides occurring in that particular position in more than 80% (uppercase letters) and 35% (lowercase letters) of analysed sequences.

The putative promoter in front of *hisD* in *C. glutamicum* AS019 identified by primer extension experiments (Jung *et al*., 2009), so far was the only known *his* promoter determined in *C. glutamicum*. The RNA-Seq technique modified for the detection of transcription start sites in *C. glutamicum* ATCC 13032 enabled the search for further *his* promoter sequences (K. Pfeifer-Sancar, A. Mentz, C. Rückert, and J. Kalinowski, manuscript in preparation). Four primary promoters were identified in front of the four his operons (P*_cg0911_*, P*_hisE_*, P*_hisH_*, P*_hisD_*). Additionally four internal promoters were observed (P*_hisN_*, P*_hisA_*, P*_hisF_*, P*_hisB_*). A −10 box hexamer fitting well to the consensus sequence TANaNT of sigma factor A (σ^A^) dependent promoters in *C. glutamicum* (Pátek and Nešvera, 2011) was determined for all four primary promoters (Fig. 3). The spacing between the transcription start site and the −10 box is 7 ± 1 nucleotides. Only the −10 box of the *hisE* promoter exhibits the TGn extension which seems to enhance promoter strength (Vasicová *et al*., 1999). The −35 boxes located 17 ± 1 nucleotides upstream of the −10 box exhibit only low similarities to the −35 consensus sequence. However, −35 boxes are generally poorly conserved in *C. glutamicum* (Pátek and Nešvera, 2011).

RNA-Seq data indicated additional internal promoters within the four histidine operons (R.K. Kulis-Horn, unpubl. data). A start of transcription was observed in front of the *hisN* gene within the *hisN*-*cg0911* operon. The data revealed a transcription start for a leaderless mRNA for *hisN* from the internal promoter P*_hisN_*. Furthermore, internal promoters were found in front the *hisA* (P*_hisA_*) and *hisF* (P*_hisF_*) genes within the *hisHA-impA-hisFI-cg2294* operon. The *hisA* gene was shown to be transcribed leaderless and *hisF* with a 5′ UTR (61 nt). A fourth internal promoter (P*_hisB_*) was observed in front of *hisB* within the *hisDCB-cg2302-cg2301* operon, resulting in a 5′ UTR (77 nt) in front of *hisB*. For all internal promoters a −10 box well-fitting the TANaNT consensus sequence is located 7 ± 1 nucleotides upstream of the transcription start site. The −35 boxes related to these promoters hardly fitted to any consensus sequence. Up to now, no data regarding the biological relevance of these internal promoters are available. However, they could play a role in regulation of histidine biosynthesis. In *S. typhimurium* and *E. coli* two internal promoters, *hisp2* and *hisp3*, were identified within the histidine operon (Grisolia *et al*., 1983; Schmid and Roth, 1983; Carlomagno *et al*., 1988). Promoter *hisp2* enables the additional transcription of the genes *his(NB)*, *hisH*, *hisA*, *hisF* and *his(IE)*, whereas *hisp3* enables transcription only of the *his(IE)* gene. Alifano and colleagues (1996) speculated that such internal promoters might reinforce the expression of distal genes in large operons to counteract the effects of natural polarity. Another function might be to temporally allow expression of some of the genes organized in an operon under specific growth conditions (Schmid and Roth, 1983). Transcription starting from *hisp2* is occluded by transcription from the primary promoter *hisp1* under histidine limited conditions, but it increases if transcription from *hisp1* does not occur at sufficient histidine supply (Alifano *et al*., 1992). In *E. coli* and *S. typhimurium* transcription from promoter *hisp1* is known to be regulated by an attenuation mechanism in response to the availability of charged histidyl-tRNAs (Kasai, 1974; di Nocera *et al*., 1978; Johnston *et al*., 1980). As transcription from the internal promoters *hisp2* and *hisp3* is not affected by this attenuation mechanism, transcription of genes from these promoters may occur even in the presence of high levels of charged histidyl-tRNA. The biological function of such a transcriptional regulation, however, still remains unexplained.

## Regulation of histidine gene expression

Regulation of biosynthetic pathways is of great importance for organisms to prevent wasting energy for the production of metabolites which are not needed under certain growth conditions. On the other hand, the regulation must also prevent the complete drainage of metabolites needed for survival and growth by temporally activating the biosynthesis. Such an accurate regulation is especially needed for the biosynthesis of amino acids as they are the building blocks of proteins and therefore needed for any enzymatic activity.

The biosynthesis of histidine is associated with high energy costs for the cell. Brenner and Ames (1971) calculated a demand of 41 ATP equivalents for the synthesis of one histidine molecule in *S. typhimurium*. Unregulated histidine biosynthesis would waste about 2.5% of the bacterial cells metabolic energy (Brenner and Ames, 1971). Based on a genome-scale stoichiometric model of the *C. glutamicum* metabolism, the ATP demand for histidine biosynthesis was calculated to be 9.4 mol_ATP_ mol_His_^−1^ (E. Zelle *et al*., pers. comm.). Since this ATP demand is the third highest for all proteinogenic amino acids exceeded only by arginine (12.0 mol_ATP_ mol_Arg_^−1^) and tryptophan (13.0 mol_ATP_ mol_Trp_^−1^), the cellular demand for a strict regulation of histidine biosynthesis is obvious.

There are three general levels of regulation of a metabolic pathway: transcriptional or translational repression, and enzyme inhibition. All three possibilities will be discussed in the following chapters.

## Transcriptional regulation

The transcriptional regulation is the first level in a regulatory cascade for metabolic pathways. Various studies concerning *E. coli* and *S. typhimurium* revealed changing mRNA levels of histidine genes with varying culture conditions (Winkler, 1996). This indicates regulation on transcriptional level, which has been also reported for *C. glutamicum* (Brockmann-Gretza and Kalinowski, 2006; Jung *et al*., 2009; 2010). The most common way of transcriptional regulation is the action of a regulatory protein binding to the operator region of a gene and thereby repressing or activating transcription (Huffman and Brennan, 2002). However, such regulatory proteins have not been identified in *S. typhimurium* or *E. coli* (Johnston *et al*., 1980). There is also no report of such a regulator in any other prokaryote, including *C. glutamicum*.

### The transcription of histidine genes is under positive stringent control

Even though no regulatory protein is involved in transcription regulation of histidine biosynthesis genes, it is addressed by the stringent response in *E. coli* and *S. typhimurium* (Winkler, 1996). The stringent response is the answer to amino acid starvation in bacteria. The effector molecules of the stringent response, guanosine tetraphosphate (ppGpp) and guanosine pentaphosphate (pppGpp), accumulate under starvation conditions (Chatterji and Ojha, 2001). On the one hand transcription of stable RNA species like tRNAs and rRNAs is repressed during Stringent Response, thereby downregulating protein synthesis. On the other hand transcription of amino acid biosynthesis genes is mostly upregulated (Chatterji and Ojha, 2001). The effector molecule (p)ppGpp is synthesized by the *relA* gene product, which catalyses phosphorylation of GDP or GTP using ATP as phosphate donor (Cashel, 1975). The *spoT* gene product was later identified to also participate in (p)ppGpp synthesis, most likely in the hydrolysis of (p)ppGpp (Laffler and Gallant, 1974; Jain *et al*., 2006). It was demonstrated for *S. typhimurium* that expression of *his* genes is stimulated 10-fold by addition of ppGpp in a *relA* deficient strain (Stephens *et al*., 1975). This stimulation is a result of enhanced transcription and not dependent on the regulatory elements needed for transcriptional attenuation (Stephens *et al*., 1975).

*Corynebacterium glutamicum* and other Actinobacteria possess a bifunctional Rel protein comprising both gene functions encoded by *relA* and *spoT* (Wehmeier *et al*., 1998; Avarbock *et al*., 1999). Analysis of a *C. glutamicum* Δ*rel* mutant, not able to synthesize (p)ppGpp, revealed a growth requirement for histidine and serine. This result suggested that transcription of histidine and serine biosynthesis genes might by positively controlled by (p)ppGpp (Tauch *et al*., 2001). The stringent response can be induced artificially by addition of the serine analogue dl-serine hydroxamate (SHX) which inhibits the seryl-tRNA synthase (Tosa and Pizer, 1971). Real-time RT-PCR analysis revealed elevated transcript levels of all histidine genes in *C. glutamicum* organized in the three operons *hisEG*, *hisHA-impA-HisFI-cg2294*, and *hisDCB-cg2302-cg2301* after treatment with SHX compared with untreated samples (Brockmann-Gretza and Kalinowski, 2006). The mRNA levels of *his* genes increased two to threefold 10 min after induction of the stringent response (Brockmann-Gretza and Kalinowski, 2006). These results clearly demonstrate that transcription of histidine biosynthesis genes is under positive stringent control in *C. glutamicum*. The *cg0911*-*hisN* operon was not identified to the time the study by Brockmann-Gretza and Kalinowski was conducted. It remains therefore unclear if this operon is also subject to positive stringent control in *C. glutamicum*.

### Transcription of histidine biosynthesis genes in *C. glutamicum* is regulated by an attenuation mechanism

Next to the global transcriptional regulation of amino acid biosynthesis genes during stringent response, transcription of histidine genes in particular is regulated by an additional mechanism in *S. typhimurium* and *E. coli*. Research on the regulation of this pathway, in addition to tryptophan biosynthesis, led to the discovery of the transcriptional attenuation mechanism (Winkler, 1996). *Escherichia coli* and *S. typhimurium* possess a leader sequence between the *hisp1* promoter and the first structural gene of the operon (Carlomagno *et al*., 1988). This leader sequence contains an open reading frame (ORF) coding for a short peptide (18 amino acids) with seven consecutive histidine residues. Transcription of the whole histidine operon is coupled to the translation of this leader peptide. During translation of the leader peptide the ribosome senses the availability of charged histidyl-tRNAs thereby influencing two possible alternative secondary structures of the nascent mRNA (Johnston *et al*., 1980). In brief, if enough charged histidyl-tRNAs are available to allow fast translation of the leader peptide, transcription of the operon is stopped due to the formation of a rho-independent terminator. On the other hand, a delay in translation due to lack of charged histidyl-tRNA promotes the formation of an anti-terminator allowing transcription of the whole operon (Johnston *et al*., 1980).

Jung and colleagues (2009) suggested a histidine-dependent transcription regulation of the *hisDCB-orf1-orf2(-hisHA-impA-hisFI)* operon in *C. glutamicum* AS019, since the corresponding mRNA was only detectable by RT-PCR if cells were grown in histidine free medium. Later, a 196 nt leader sequence in front of *hisD* was identified (Jung *et al*., 2010). Since no ORF coding for a short peptide containing several histidine residues is present in this leader sequence, a translation-coupled transcription attenuation mechanism like in *E. coli* and *S. typhimurium* can be excluded. Instead, a T-box mediated attenuation mechanism controlling the transcription of the *hisDCB-orf1-orf2(-hisHA-impA-hisFI)* operon has been proposed (Jung *et al*., 2010). Computational folding analysis of the 196 nt 5′ UTR from *C. glutamicum* AS019 revealed two possible stem-loop structures. In the first structure, the terminator structure, the SD sequence (−10 to −17 nt; numbering relative to *hisD* translation start site) is sequestered by formation of a hair pin structure. In the second structure, the anti-terminator structure, the SD sequence is available to ribosomes. Additionally, a histidine specifier CAU (−92 to −94 nt) and the binding site for uncharged tRNA 3′ ends UGGA (−58 to −61 nt) were identified. All these components are characteristics of T-box RNA regulatory elements. T-box RNAs are members of riboswitch RNAs commonly modulating the expression of genes involved in amino acid metabolism in Gram-positive bacteria (Gutierrez-Preciado *et al*., 2009). They were first discovered in *B. subtilis* regulating the expression of aminoacyl-tRNA synthases (Henkin, 1994). Uncharged tRNAs are able to concurrently bind to the specifier sequence and the UGGN-sequence of the T-box RNA via the tRNAs anti-codon loop and free CCA-3′ end, respectively, thereby influencing the secondary structure of the mRNA (Vitreschak *et al*., 2008). The T-box mechanism results in premature transcription termination due to the formation of a rho-independent transcription terminator hairpin structure in the absence of uncharged tRNAs (Henkin, 1994). Jung and colleagues (2010) showed that chloramphenicol acetyltransferase (CAT) activity decreases in response to histidine in the medium if the 196 nt 5′ UTR in front of *hisD* is transcriptionally fused to the chloramphenicol acetyltransferase (*cat*) gene, demonstrating its transcription termination ability. Additionally, the replacement of the UGGA sequence (−58 to −61 nt) reduced specific CAT activity even in the absence of histidine, strongly supporting the involvement of uncharged tRNAs in the regulatory mechanism (Jung *et al*., 2010).

To test the effect of histidine on the transcription of the remaining *his* operons we conducted real-time RT-PCR analysis of *C. glutamicum* ATCC 13032 grown on minimal medium without and with 1 mM histidine supplementation. Surprisingly, these experiments did not reveal any differences in transcription of *his* genes, neither organized in the *hisDCB-cg2302-cg2301* operon nor in the three remaining operons (data not shown). The same holds true for a histidine dipeptide addition experiment analysed via subsequent real-time RT-PCR and micro-array analysis. No differences in transcription of *his* genes were observed after addition of the dipeptide, suggesting that, in contrast to *C. glutamicum* AS019, histidine does not affect transcription of the *his* genes in *C. glutamicum* ATCC 13032 (data not shown). The inconsistent results for the two *C. glutamicum* strains cannot be explained so far. Although the DNA-sequence upstream of the *hisD* seems to be identical in both *C. glutamicum* strains, it cannot the excluded completely that transcription of *his* genes is regulated differently in these two strains in respect to the effect of histidine. Strain-specific differences are already obvious in the different transcription start sites in front of the *hisD* genes in both organisms resulting in 5′ UTRs of different length. The *hisD* leader sequence in *C. glutamicum* ATCC 13032 is much shorter and consists of only 93 nt (R.K. Kulis-Horn, unpubl. obs.). Whereas the longer *hisD* 5′ UTR from *C. glutamicum* AS019 is clearly involved in transcriptional regulation, we suggest a translational control mechanism for the shorter *hisD* 5′ UTR from *C. glutamicum* ATCC 13032, which will be discussed in detail below.

## Translational regulation

The translation process is part of the attenuation mechanism regulating transcription of *his* genes in *E. coli* and *S. typhimurium* (see above). Besides this, there is no report that translation of the *his* operon is regulated once the complete mRNA is transcribed in these two organisms. However, there is evidence for a translational regulation mechanism in *C. glutamicum*.

Jung and colleagues (2010) identified a 196 nt long 5′ UTR in front of the *hisDCB-orf1-orf2* operon in *C. glutamicum* AS019 which can fold into two alternative secondary structures, one of them sequestering the SD sequence in a hair pin (see above). Although Jung and colleagues (2010) demonstrated that this T-box like RNA-element affects transcription of the *hisDCB-orf1-orf2* operon, the possibility of an additional translational control by this process remains. T-box RNAs are usually known to regulate transcription only (Gutierrez-Preciado *et al*., 2009). However, a comparative analysis of RNA regulatory elements of amino acid metabolism genes from various bacteria revelled that some T-boxes from Actinobacteria might be involved in regulation of translation initiation instead (Seliverstov *et al*., 2005). The T-box RNA identified in front of the *ileS* gene of *C. glutamicum* for example sequesters the SD sequence in the alternative hairpin instead of forming a transcriptional terminator (Seliverstov *et al*., 2005). Yet, the bioinformatics analysis performed by Seliverstov *et al*. did not identify similar regulatory RNA elements in front of the *his* genes in *C. glutamicum* or other Actinobacteria. This might be attributed to the fact that translation-regulating T-boxes of Actinobacteria have a slightly different structure as compared with classical T-boxes. They are shorter and the specifier codon is located in the loop of the specifier hairpin and not in the bulge (Vitreschak *et al*., 2008). The RNA element in front of the *hisDCB-orf1-orf2* proposed by Jung and colleagues (2010) is shorter as compared with classical T-boxes, since two of the conserved stems (stem II and III) are missing. For this reason, however, it is even much shorter than other known T-box RNAs from Actinobacteria. Moreover, the ‘T-box-sequence’, the 14 most highly conserved residues in T-box RNAs (Gutierrez-Preciado *et al*., 2009), is only moderately conserved and the histidine specifier is present in a bulge of the specifier hairpin and not in its loop. Consequently, the structure of this regulatory RNA element does not fit the characteristics of neither classical nor short T-box RNAs. It remains ambiguous if this RNA element really represents a T-box or another type of riboswitch.

**Fig. 4:**
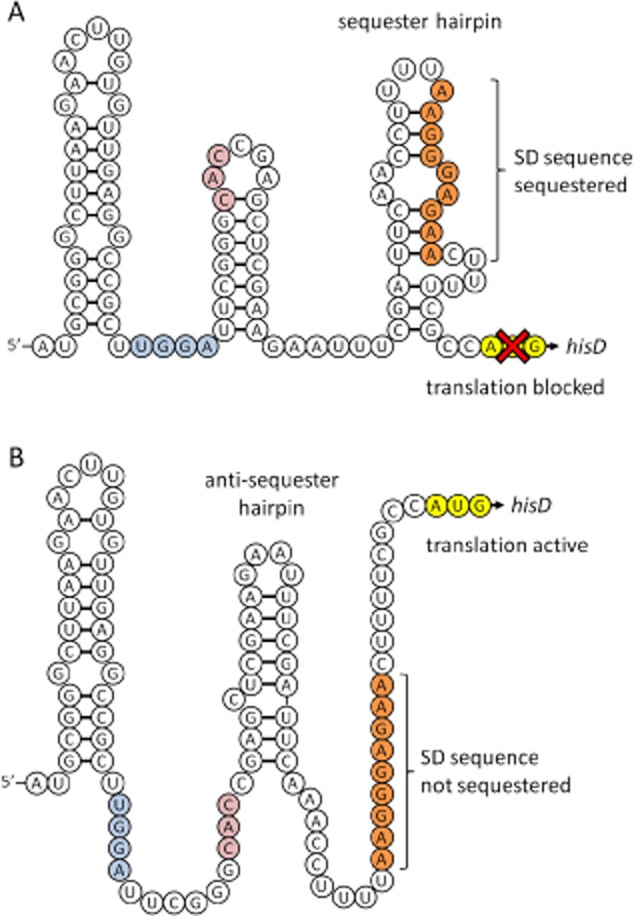
Secondary structure model of the 5′ UTR of the *hisDCB-cg2302-cg2301* mRNA from *C. glutamicum* ATCC 13032. Nucleotides shown in orange and yellow represent the SD sequence and the *hisD* start codon respectively. The histidine specifier (CAC) is shown in red and the putative CCA binding site for uncharged tRNA 3′ ends (UGGA) is shown in blue. Both sequences might be involved in a histidyl-tRNA dependent riboswitch mechanism.A. SD sequester structure. The SD sequence is sequestered in a hairpin and not available to ribosomes. Translation of the *hisD* gene is blocked.B. SD anti-sequester structure. The formation of the anti-sequester hairpin prevents the formation of the sequester hairpin. The SD sequence is available to ribosomes and *hisD* is translated. Uncharged histidyl-tRNA interacting with the histidine specifier and the CCA binding site might be involved in the stabilization of the anti-sequester hairpin, resulting in a switch from the SD sequester to the SD anti-sequester structure.

We identified an even shorter 5′ UTR in front of the *hisDCB-cg2302-cg2301* operon in *C. glutamicum* ATCC 13032 by means of RNA-Seq (see above). This 5′ UTR is only 93 nt in length. Like the 196 nt 5′ UTR identified in *C. glutamicum* AS019 (Jung *et al*., 2010), this short 5′ UTR is able to fold into two alternative secondary structures, one of them sequestering the SD sequence (Fig. 4). The binding site for uncharged tRNA 3′ ends UGGA (−58 to −61 nt; numbering relative to *hisD* translation start site) is present in this structure, too. However, the histidine specifier CAU described by Jung and colleagues (2010) is already located upstream of the transcribed region in *C. glutamicum* ATCC 13032 and is therefore not part of its 5′ UTR. But there is another histidine specifier CAC (−49 to −51, numbering relative to *hisD* translation start site) present in this short 5′ UTR. This CAC histidine specifier seems to be even better suited for interaction with histidyl-tRNAs as it is exactly complementary to the GUG anti-codon of the single histidyl-tRNA in *C. glutamicum* (Kalinowski *et al*., 2003). There is also a strong bias towards the presence of a C in the third position of the specifier sequence of most T-box RNAs (Gutierrez-Preciado *et al*., 2009). Nevertheless, a T-box regulatory mechanism seems to be even more unlikely than in the case of *C. glutamicum* AS019. The histidine specifier and the binding site for uncharged tRNA 3′ ends are separated by only seven nucleotides. The interaction of both binding sites with one histidyl-tRNA molecule at the same time seems to be improbable, if not impossible. Moreover, in all known T-box RNAs the specifier is always located upstream of the binding site for uncharged tRNA 3′ ends and not downstream as in this case (Vitreschak *et al*., 2008; Gutierrez-Preciado *et al*., 2009). Therefore, a T-box regulatory mechanism seems unlikely. However, it is still possible that histidyl-tRNAs function as effectors in another type of riboswitch mechanism, since elements for binding of histidyl-tRNAs are present and two alternative secondary structures are predicted. The sequestration of the SD sequence within a hairpin in one of these structures, together with the observation that histidine does not affect the transcription of *his* genes (see above), suggests a translational regulatory role of the 5′ UTR in front of *hisDCB-cg2302-cg2301* operon in *C. glutamicum* ATCC 13032. Such a translational control would primary affect the expression of the *hisD* gene itself, since SD sequences are present in front of all genes in the operon. As long as these SD sequences are not sequestered by additional secondary mRNA structures, *de novo* initiation of translation of all downstream genes should still be possible even if *hisD* is not translated (Yoo and RajBhandary, 2008). Since HisD catalyses the final steps of histidine biosynthesis, the translational regulation of only this particular enzyme would enable a very rapid recovery of biosynthesis if histidine becomes limiting.

## Enzymatic regulation

The gene product of *hisG*, the ATP phosphoribosyltransferase (HisG), is the most important enzyme being regulated on enzymatic level in histidine biosynthesis. This enzyme catalyses the first step of histidine biosynthesis, the condensation of ATP and PRPP to PR-ATP. The regulation of this particular enzyme is of remarkable importance, as it prevents waste of ATP and also of PRPP. The latter is not only the substrate for the biosynthesis of histidine, but also used for the *de novo* synthesis of purines (Zhang *et al*., 2008) and pyrimidines (Garavaglia *et al*., 2012), the tryptophan biosynthesis (Sprenger, 2007), and for the synthesis of arabinogalactan, an important component of the corynebacterial cell wall (Alderwick *et al*., 2006).

### HisG is affected by feedback inhibition in *C. glutamicum*

It has been demonstrated very early that HisG from *S. typhimurium* (HisG*_St_*) is subject to histidine-mediated feedback inhibition in a non-competitive manner (Martin, 1963a) and the same holds true for HisG from *E. coli* (HisG*_Ec_*) (Winkler, 1996). It has been suggested that ATP-PRT from *C. glutamicum* (HisG*_Cg_*) is subject to histidine-mediated feedback inhibition, too, since the histidine analogues 2-thiazolyl-dl-alanine (2-TA) and 1,2,4-triazolyl-3-alanine (TRA) inhibit growth of *C. glutamicum* (Araki and Nakayama, 1971). These two analogues are known to be non-competitive inhibitors of ATP-PRT in *S. typhimurium* (Martin, 1963a). Analogue-resistant *C. glutamicum* mutants isolated by Araki and Nakayama (1971) accumulate histidine in the supernatant, indicating that these mutants are deregulated in histidine biosynthesis most likely due to loss of feedback inhibition. Later, by performing enzyme assays with cell-free extracts it was demonstrated that HisG*_Cg_* is indeed inhibited by l-histidine (Araki and Nakayama, 1974), and recently, Zhang and colleagues (2012) confirmed the inhibition by histidine on the purified HisG*_Cg_* enzyme. Histidine acts as non-competitive inhibitor of HisG*_Cg_* having a K_i_ value of 0.11 ± 0.02 mM (Zhang *et al*., 2012). The enzyme is inhibited stronger by histidine than the corresponding ATP-PRTs from *Thermotoga maritima*, but less than those from *S. typhimurium* and *L. lactis* (Zhang *et al*., 2012). It was also demonstrated that, like in *S. typhimurium* (Martin, 1963a; Morton and Parsons, 1977a), AMP and ADP are competitive inhibitors with respect to ATP with K_i_ values of 1.29 ± 0.42 mM and 0.88 ± 0.35 mM respectively (Zhang *et al*., 2012). The inhibitory effect of these two substances with respect to PRPP was not tested. The inhibition of ATP-PRT by AMP and ADP enables to stop the highly energy-demanding histidine biosynthesis if the cells overall energy status is low.

d-Histidine and the histidine intermediates IGP, IAP, Hol-P, l-histidol, and l-histidinal show no inhibitory effect on HisG*_St_* (Martin, 1963a), indicating that HisG inhibition is very specific. l-Histidine itself inhibits both, HisG*_St_* and HisG*_Cg_*, only as dipolar ion with a positively charged α-amino group, since the inhibitory effect is abolished under alkaline pH conditions (Martin, 1963a; Zhang *et al*., 2012). It is known from studies with *S. typhimurium* that ppGpp enhances the inhibitory effect of histidine, resulting in complete inhibition of enzyme activity already at moderate histidine concentrations (Morton and Parsons, 1977b). The alarmone ppGpp accumulates during general amino acid starvation and positively effects *his* operon transcription (see above). Therefore, the synergetic inhibition of HisG*_St_* by ppGpp and histidine prevents unneeded histidine biosynthesis during stringent response induced by an amino acid different from histidine (Winkler, 1996). Since transcription of *his* genes in *C. glutamicum* is induced during stringent response, a synergetic inhibitory effect of ppGpp and l-histidine on HisG*_Cg_* might exist, too, but has never been tested.

Gel filtration experiments with HisG*_Cg_* demonstrated that it exists in a dimeric and a hexameric form (Zhang *et al*., 2012). It is already known for the highly similar HisG*_Mt_* that it exists as homodimer in the absence of histidine and at low enzyme concentrations, but it forms hexamers or higher oligomers in the presence of histidine (Cho *et al*., 2003). This is in accordance with data obtained with HisG*_Ec_*, whose dimer represents the active form of the enzyme whereas higher oligomers are inactive (Tébar *et al*., 1973). Due to the high structural similarity (Zhang *et al*., 2012) it is very likely that HisG*_Cg_* acts in the same way, i.e. active in its dimeric form and inactive in a histidine-induced hexamer form. The histidine-induced change in quaternary structure from a dimeric to a hexameric form of HisG*_Ec_* can be reversed by addition of the substrate PRPP (Tébar *et al*., 1973). This might also by true for HisG*_Cg_* since the inhibitory effect of histidine is reduced by excess of PRPP (Araki and Nakayama, 1974). According to a predicted structure model, HisG*_Cg_* monomers are L-shaped and composed of three distinct domains (Zhang *et al*., 2012). The first two domains are the catalytic domains and the third domain is able to bind histidine and therefore is regarded to be the regulatory domain (Cho *et al*., 2003; Zhang *et al*., 2012). It is known from the highly similar HisG*_Mt_* that one histidine molecule interacts with residues of two different domains III at the same time. This led to the suggestion that direct interaction with histidine is responsible for aggregation of three active HisG dimers to one inactive hexamer (Cho *et al*., 2003). Binding of AMP additionally establishes intersubunit interactions that stabilize the histidine-bound HisG*_Mt_* hexamer (Cho *et al*., 2003). This effect of AMP on HisG quaternary structure is also known for *E. coli*, where the same hexamer-stabilizing effect is observed additionally with the product PR-ATP and high enzyme concentrations (Klungsöyr and Kryvi, 1971). The crystal structure of HisG*_Ec_* in complex with AMP demonstrated that AMP is not binding to the ATP binding site. Instead, the monophosphate and ribose moieties of AMP are binding to the PRPP binding site and only the adenine ring occupies the ATP binding site (Lohkamp *et al*., 2004), which explains the surprising observation that AMP acts as a competitive inhibitor with respect to both substrates ATP and PRPP (Morton and Parsons, 1977a).

The crystal structure of HisG*_Mt_* in complex with histidine enabled the identification of amino acid residues interacting with histidine (Cho *et al*., 2003). These residues correspond to Gln215, Leu231, Thr235, Met250, and Ala270 in HisG*_Cg_*. The role of the residues Gln215, Leu231, Thr235, and Ala270 in feedback inhibition of HisG*_Cg_* has been confirmed by mutation studies, recently (Zhang *et al*., 2012). A mutated enzyme with three simultaneous substitutions (N215K/L231F/T235A) turned out to be least inhibited by histidine in this study. The mutated enzyme showed a K_i_ of 4.15 ± 0.21 mM without decreasing the specific enzyme activity (Zhang *et al*., 2012).

To conclude, the HisG enzyme performing the first step in histidine biosynthesis is the only known enzyme in the biosynthesis that is controlled by a feedback inhibition mechanism involving the end-product of the biosynthesis, l-histidine. Therefore, it constitutes the most interesting target in the development of histidine-producing *C. glutamicum* strains.

## l-Histidine uptake

Uptake systems for l-histidine are known from several microorganisms. *Escherichia coli* and *S. typhimurium* possess two main uptake systems, the high affinity HisJQMP ABC transport system specific for histidine and the low affinity AroP system transporting all aromatic amino acids (Winkler, 1996). The K_m_ values for histidine transport in *S. typhimurium* are 8 × 10^−8^ M and 10^−4^ M for the HisJQMP permease and the AroP-system respectively (Ames and Roth, 2005). In *B. subtilis* the histidine-specific transporter is encoded by *hutM* as a part of the histidine utilization operon (Yoshida *et al*., 1995).

### l-Histidine uptake system in *C. glutamicum* is encoded by *cg1305*

*Corynebacterium glutamicum* possesses a general uptake system for aromatic amino acids encoded by the gene *aroP* (Wehrmann *et al*., 1995). The transporter is able to import the three aromatic amino acids tyrosine, tryptophan, and phenylalanine. But, unlike the corresponding transporter from *E. coli* and *S. typhimurium* (Ames and Roth, 2005), it is unable to import histidine (Wehrmann *et al*., 1995). Therefore, a histidine uptake system in *C. glutamicum* still remains unidentified. Since the gene *cg2301* exhibits characteristics of a transporter and is part of the *hisDCB-cg2302-cg2301* operon, it can be regarded as a candidate to encode a l-histidine uptake system. However, the deletion of *cg2301* did not affect growth of a histidine-auxotrophic Δ*hisG* mutant in minimal medium supplemented with histidine, demonstrating still functional histidine uptake (R.K. Kulis-Horn, unpubl. obs.). Further candidates for encoding the unknown l-histidine uptake system in *C. glutamicum* are the genes *cg1305*, *cg0555*, and *aroP*, since the amino acid sequence of the histidine transporter HutM of *B. subtilis* shows the highest similarity to their deduced amino acid sequences. The gene *cg1305* has been recently reported to encode the l-phenylalanine-specific transporter (Zhao *et al*., 2011) and the gene product of *cg0555* has been characterized as γ-aminobutyric acid uptake system (Zhao *et al*., 2012). Since deletion of *aroP* did not affect growth of a histidine auxotrophic Δ*hisG* mutant on minimal medium supplemented with histidine (R.K. Kulis-Horn, unpubl. obs.), the gene product of *aroP*, confirming the results of Wehrmann and colleagues (1995), does not encode the histidine uptake system in *C. glutamicum*. The same holds true for *cg0555*, since a deletion had no effect on growth of the Δ*hisG* mutant (R.K. Kulis-Horn, unpubl. obs.). The deletion of *cg1305*, however, resulted in a strongly reduced growth rate of the histidine auxotrophic mutant already on complex medium and growth of this mutant was almost completely inhibited on minimal medium supplemented with histidine (R.K. Kulis-Horn, unpubl. obs.). These results strongly suggest that *cg1305* encodes a histidine uptake system, and probably that it is the only histidine importer in *C. glutamicum*. Recently, ^14^C-labelling experiments demonstrated that the transporter encoded by *cg1305* is able to import l-phenylalanine (Zhao *et al*., 2011). Additionally, the uptake of labelled l-tyrosine, l-tryptophan, and l-proline was tested in this study, but does not occur via this transporter. The ability of importing labelled l-histidine was not tested, but strikingly unlabelled l-histidine does not compete with the uptake of labelled l-phenylalanine (Zhao *et al*., 2011). This surprising result is somehow inconsistent with our finding that *cg1305* encodes the only histidine uptake system in *C. glutamicum*, since one would expect that unlabelled histidine slows down the uptake of labelled phenylalanine. A possible explanation is the existence of several uptake systems for l-phenylalanine in *C. glutamicum* (Cg1305, AroP, and at least one additional unknown) (Zhao *et al*., 2011). Although Zhao and colleagues (2011) used a Δ*aroP* strain in their study, the unknown third l-phenylalanine transporter might counteract the reduced phenylalanine uptake via Cg1305 in the presence of histidine, assuming that the unknown transporter does not additionally import histidine. Since our results with the *C. glutamicum* Δ*hisG* Δ*cg1305* did not indicate additional l-histidine uptake systems beside Cg1305, our observation and the results from Zhao *et al*. might still be consistent. However, the uptake of labelled l-histidine should be tested to undoubtedly confirm that *cg1305* encodes the l-histidine uptake system in *C. glutamicum*.

## l-Histidine export

To our knowledge no histidine export system has been described in any organism. Exporters for other amino acids, however, are well known in *E. coli* and *C. glutamicum*, including efflux systems for l-lysine, l-arginine, l-threonine, l-cysteine, l-leucine, l-isoleucine, and l-valine (Eggeling and Sahm, 2003). Hashimoto *et al*. recently showed that l-glutamate, l-aspartate and l-phenylalanine are secreted via a mechano-sensitive channel by passive diffusion in *C. glutamicum* (Hashimoto *et al*., 2012). In the past, the export of amino acids by bacteria was believed to be an artificial result of industrial overproduction and to have no biological relevance. But, next to regulation of the biosynthesis of an amino acid and degradation, the corresponding export might be an important possibility to maintain amino acid homoeostasis, especially in peptide-rich environments (Eggeling and Sahm, 2003). Genes for histidine utilization, which are present in several pathogenic *Corynebacterium* species, are missing in *C. glutamicum* (Schröder *et al*., 2012). However, Bellmann and colleagues (2001) demonstrated the ability of *C. glutamicum* to export histidine, which may allow to maintain histidine homoeostasis in an environment rich in histidine-containing peptides. Addition of 2 mM His-Ala dipeptide to a *C. glutamicum* culture resulted in a steady increase of external histidine concentration (Bellmann *et al*., 2001). The export, however, seems to be rather inefficient as internal histidine concentration rises from zero to 200 mM after addition of the dipeptide (Bellmann *et al*., 2001). Since *C. glutamicum* does not secrete any peptidases (Erdmann *et al*., 1993), the only explanation for the rising external histidine concentration is export of histidine that was cleaved of from the dipeptide itracellularly. However, no candidate gene encoding the exporter has been proposed so far. Interestingly, histidine acts as a co-inducers of *lysE* transcription, a gene encoding the l-lysine and l-arginine efflux system in *C. glutamicum*, although histidine is not exported by LysE (Bellmann *et al*., 2001). There is no explanation, why histidine acts as co-inducer of the exporter, which is unable to export l-histidine. In fact, this might cause a disadvantageous situation for the cell as high histidine concentrations might trigger efflux of l-lysine and l-arginine although their concentrations are low. This negative effect, however, might somehow be counteracted by the high K_m_ value of 20 mM for l-lysine export (Bröer and Krämer, 1991).
